# Vibrational control of the reaction pathway in the H + CHD_3_ → H_2_ + CD_3_ reaction

**DOI:** 10.1126/sciadv.abm9820

**Published:** 2022-03-30

**Authors:** Roman Ellerbrock, Bin Zhao, Uwe Manthe

**Affiliations:** 1 Theoretische Chemie, Fakultät für Chemie, Universität Bielefeld, Universitätsstr. 25, D-33615 Bielefeld, Germany.; 2 Department of Chemistry and The PULSE Institute, Stanford University, Stanford, CA 94305, USA.; 3 SLAC National Accelerator Laboratory, Menlo Park, CA 94025, USA.

## Abstract

An accurate full-dimensional quantum state-to-state simulation of the six-atom title reaction based on first-principles theory is reported. Counterintuitive effects are found: Increasing the energy in the reactant’s CD_3_ umbrella vibration reduces the energy in the corresponding product vibration. An in-depth analysis reveals the crucial role of the effective dynamical transition state: Its geometry is controlled by the vibrational states of the reactants and subsequently controls the quantum state distribution of the products. This finding enables generalizing the concept of transition state control of chemical reactions to the quantum state–specific level.

## Teaser

Reactant vibrational excitation modifies the transition state geometry and thereby controls the product distribution.

## INTRODUCTION

The quantum state–specific understanding of chemical reactions is a central objective of research connecting chemistry to the fundamental physical laws of nature. While this aim has largely been achieved for triatomic and simple tetraatomic reactions, polyatomic reactions with a large number of interacting degrees of freedom still pose a formidable challenge. Reactions of methane with atoms, important processes in atmospheric and combustion chemistry, are studied at the forefront of research aiming to extend the boundaries toward larger systems. Impressive experiments provided very detailed information about reactions with chlorine and fluorine (*1*–*7*). Accurate full-dimensional quantum dynamics simulations investigated the correlation between the quantum state of reactants and products for the H + CH_4_ → H_2_ + CH_3_ reaction (*8*). However, quantum theory at the state-to-state level for the reaction of methane with chlorine recently relied on a reduced dimensional model considering seven active degrees of freedom (*7*).

The full quantum state–resolved description of a polyatomic reaction provides an enormous wealth of data, and theory plays a key role in the data analysis, model development, and interpretation (*9*–*11*). Using numerically exact methods and efficient algorithms, modern first-principles theory has achieved predictive power and can, under favorable circumstances, be as accurate and reliable as experimental measurements (*12*–*14*). Here, predictive numerical calculations based on accurate first-principles theory are used to provide a reactant and product quantum state–resolved description of the H + CHD_3_ → H_2_ + CD_3_ reaction and to develop a detailed understanding of the underlying reaction dynamics. The emerging picture will connect the observed vibrational control of the reaction products to established concepts of the transition state control of chemical reactions.

Various full- and reduced dimensional quantum dynamics calculations studied initial state-selected reaction probabilities and cross sections for reactions of methane with different atoms (*15*–*27*). By extending the ideas encoded in the Polanyi rules (*28*), which originally emerged from the analysis of atom-diatom reactive scattering, the observed vibrational control of the chemical reactivity could be explained by differences between the vibrational modes in the reactants and at the transition state (*20*, *23*, *29*–*31*). Full-dimensional state-to-state calculations for the H + CH_4_ → H_2_ + CH_3_ reaction (*8*) observed the “loss of vibrational memory” in the product state distributions: At total energies close to the barrier energy, the vibrational state distribution of the products is approximately independent of the vibrational states of the reactants. Subsequent work on various tetraatomic reactions (*32*–*34*) demonstrated the ubiquitous nature of the loss of memory effect. However, methane is a highly symmetric molecule with delocalized vibrational modes. The nonlocal character of the vibrations and the presence of multiple degenerate components prohibit the localization of the vibrational energy in specific bonds or individual bending angles. The resulting averaging might enhance the loss of memory effect and hide more subtle correlations between the quantum states of the reactants and products.

On the basis of the first full-dimensional state-to-state quantum dynamics calculations studying the reaction of isotopically substituted methane, we will show that specific correlations between the reactant and product vibrational states can persist at energies close to the barrier height. Considering an isotopically substituted CHD_3_ reactant instead of the highly symmetric CH_4_, vibrational energy can be deposited in the localized CH stretching or the CD_3_ umbrella bending vibrations, and its effect on the product state distributions can be studied selectively.

## RESULTS

**Fig. 1.**
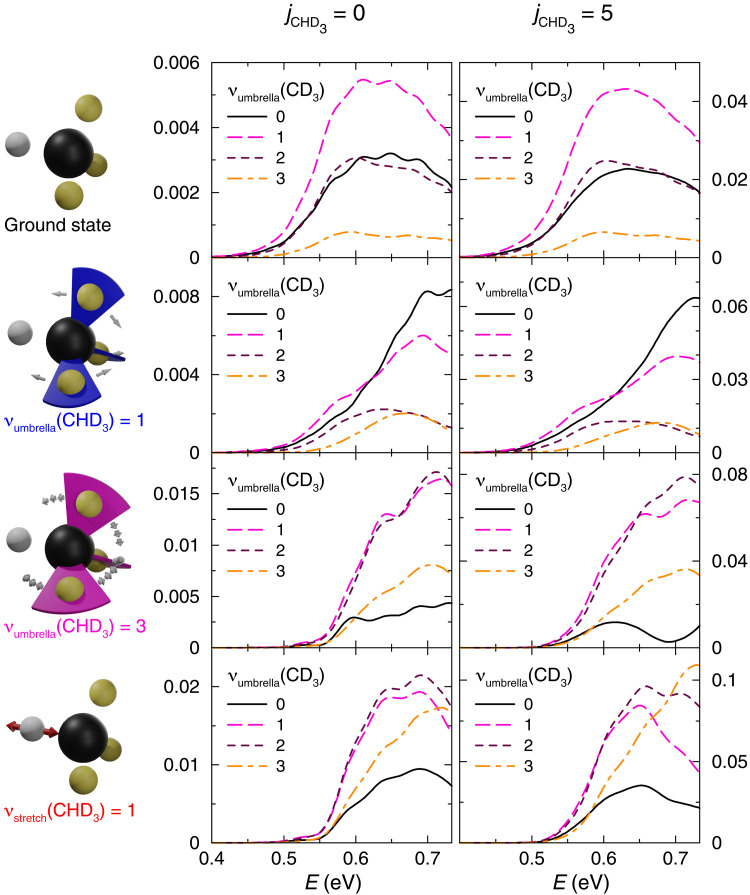
State-to-state reaction probabilities. H + CHD_3_ → H_2_ + CD_3_ state-to-state reaction probabilities (*J* = 0) depending on the different quantum states of the CHD_3_ reactant and the CD_3_ product molecules are displayed as a function of the total energy (measured relative to the ground state energy of the separated reactants). The formed products show only excitation in the CD_3_ umbrella bending vibration, and the corresponding reaction probabilities are displayed by different lines in each panel. Probabilities for reaction from different ro-vibrational states of the CHD_3_ reactant molecule are given in the different panels. The left and right columns refer to reactants showing the rotational quantum numbers *j*_CHD_3__ = 0 and *j*_CHD_3__ = 5 (averaging with respect to degenerate components). The rows refer to the different vibrational states of the reactants discussed in the text (schematic images of the respective vibrational motion are depicted).

H + CHD_3_ → H_2_ + CD_3_ state-to-state reaction probabilities for vanishing total angular momentum (*J* = 0) and CHD_3_ reactants in various ro-vibrational states are shown in Fig. 1. The final vibrational state of the products is analyzed without resolving the rotational states of the H_2_ and CD_3_ product molecules, and the reaction probabilities are averaged with respect to the orientation-dependent reactant rotational quantum number *m*. Vibrational excitation is observed only in the CD_3_ umbrella bending mode, a finding in line with previous results obtained for the H + CH_4_ → H_2_ + CH_3_ reaction and an interpretation that assumes a sudden decay of the activated complex determining the product state distribution: The CD_3_ umbrella bending mode is the only low-frequency vibrational mode in the products that is strongly coupled to the reaction coordinate. In the subsequent analysis, the product state distribution in this mode, i.e., the relative amount of products formed in the different vibrational states for a given initial condition, is used as a probe that provides important insights into the state-to-state reaction dynamics. Comparing results for CHD_3_ reactants in the rotational states *j* = 0 (left-hand side of Fig. 1) and *j* = 5 (right-hand side of Fig. 1), the product vibrational state distributions are found to be independent of the rotational state of the reactants. However, the product state distributions resulting from different initial vibrational states of the CHD_3_ reactant (shown in different rows in Fig. 1) vary significantly even at energies close to the threshold energy. This finding is in apparent contradiction with the “loss of vibrational memory” principle.

To study the effect in detail, vibrational excitations of the reactants that most strongly promote the reactivity (*35*) are considered: the vibrational ground state, the first C─H stretch excited state [ν_stretch_(CHD_3_) = 1], and the first CD_3_ umbrella bending excited state [ν_umbrella_(CHD_3_) = 1] of the CHD_3_ molecule. The importance of these states can be straightforwardly rationalized by a comparison of the reactant and transition state normal modes. Furthermore, the third CD_3_ umbrella bending state [ν_umbrella_(CHD_3_) = 3] shows unusually high reactivity, which results from reactivity borrowing caused by Fermi resonance–type state mixing with the ν_stretch_(CHD_3_) = 1 state. Marked differences between the product state distributions displayed in the different rows can be observed. ν_umbrella_(CHD_3_) = 1 reactants yield products showing the lowest amount of CD_3_ umbrella bending excitation in the CD_3_ product, while ν_stretch_(CHD_3_) = 1 reactants yield products with the highest one. This seemingly counterintuitive result indicates a substantial vibrational energy redistribution during the reaction processes. The product state distribution obtained for ν_umbrella_(CHD_3_) = 3 reactants roughly equals the one obtained for ν_stretch_(CHD_3_) = 1 reactants, an expected consequence of the Fermi resonance–type state mixing (*35*).

## DISCUSSION

**Fig. 2.**
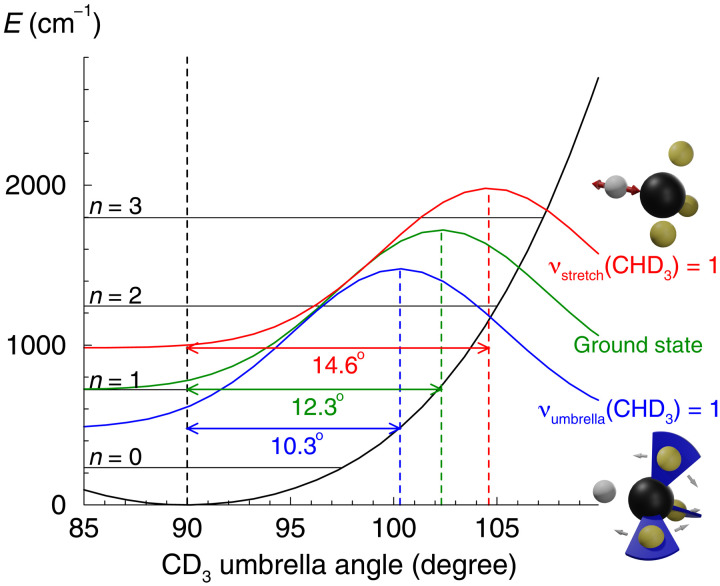
Effective transition state geometries from product state distributions. Transition state wave packets are reconstructed from the computed product state distributions in the CD_3_ umbrella bending mode using the sudden approximation. The CD_3_ product’s potential as a function of the umbrella angle (in harmonic approximation) and the corresponding vibrational levels are indicated by black lines. Transition state wave packets corresponding to reaction of CHD_3_ reactants in ground vibrational state, the umbrella bending excited state [ν_umbrella_(CHD_3_) = 1], and the C─H stretch excited state [ν_stretch_(CHD_3_) = 1] are displayed by green, blue, and red lines, respectively. The corresponding product state distributions shown in Fig. 1 peak at ν_umbrella_(CD_3_) values of 1, between 0 and 1, and between 1 and 2, respectively. These values relate to Huang-Rhys parameters of $\sqrt{2}$, 1, and 2, respectively, and coordinate displacements of 12. 3^∘^, 10. 3^∘^, and 14. 6^∘^ (see the Supplementary Materials for more details).

**Fig. 3.**
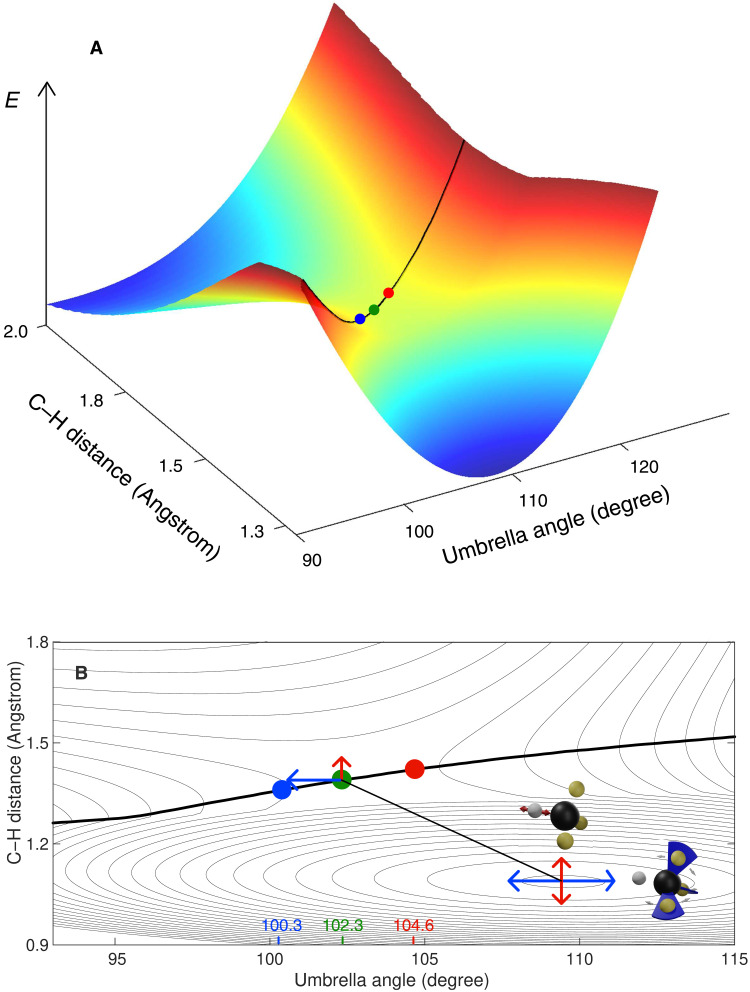
Vibrational control of the effective transition state geometry. The dependence of the effective dynamical transition state on the quantum state of the CHD_3_ reactant is illustrated. In (**A**), the potential energy surface of the H + CHD_3_ → H_2_ + CD_3_ reaction is shown as a function of the C─H stretching and CD_3_ umbrella bending coordinate (optimizing the potential with respect to all other coordinates). The ridge separating reactant and product geometries is highlighted by a black line, and the different effective transition state geometries are indicated as colored dots (umbrella angles and color coding are taken from Fig. 2). In (**B**), a contour plot of the same potential energy surface is shown. Here, the effect of vibrational excitation on the geometry of the effective transition state is illustrated. The blue and red arrows mark the (additional) amplitude related to one quantum of excitation in the umbrella bending and C─H stretching modes, respectively.

The unexpected product state distributions are a result of the vibrational control of the effective dynamical transition state: the geometry of the dynamic transition state is not exclusively defined by the saddle point of the potential energy surface but also affected by the quantum state of the reactants. To derive this conclusion, we first obtain the geometry of the effective transition state from the computed product state distribution (Fig. 2). Then, the resulting initial state-dependent effective transition state geometries are rationalized, considering the specific vibrational excitations of the reactants (Fig. 3). We expect that these conclusions can be extended from reaction probabilities to scattering cross sections. Several related studies (*17*, *18*, *24*, *25*) already established that vibrational excitation affects reaction probabilities for *J* = 0 and integral cross sections in the same way. The subsequent discussion focuses exclusively on product state distributions because the initial state-selected reaction probabilities, which are equal to the state-to-state reaction probabilities summed over all product states, were already discussed previously (*35*–*37*).

The transition state or activated complex plays a key role in a chemical reaction proceeding via a potential barrier. It is the crucial bottleneck that has to be passed on the way from reactants to products. In the H + CHD_3_ → H_2_ + CD_3_ reaction, the dynamics from the transition state toward the products essentially is a downhill motion. Thus, the product state distributions can be rationalized using the sudden approximation (*38*) that directly maps an initial wave packet onto product states, assuming an instantaneous decay. The initial wave packets in the CD_3_ umbrella bending coordinate are reconstructed from the computed product state distributions using the sudden approximation and a simple harmonic oscillator model. As shown in Fig. 2, product state distributions for reaction from the ground state, the ν_umbrella_(*CD*_3_) = 1 state, and the ν_stretch_(CHD_3_) = 1 state of CHD_3_ are associated with transition state wave packets centered around CHD_3_ umbrella angles of 102. 3^∘^, 100. 3^∘^, and 104. 6^∘^, respectively. This analysis suggests that the geometry of the activated complex or effective dynamical transition state depends on the reactant’s vibrational excitation. Vibrational excitation in the umbrella bending mode of the CHD_3_ reactant yields a smaller, more product-like CD_3_ umbrella bending angle in the effective transition state, while vibrational excitation in the reactant’s C─H stretching mode results in a larger, more reactant-like CD_3_ umbrella bending angle.

To explain this finding, the dependence of the potential energy on the C─H distance and the CD_3_ umbrella bending angle is depicted in Fig. 3. The ridge in the potential energy landscape separating the reactant and product regions is clearly visible in the three-dimensional plot. Colored dots mark the different effective transition state geometries discussed above. The larger umbrella angle observed for C─H stretching excited reactants correlates with a larger C─H distance in the effective transition state. Thus, depositing energy in a vibration mode results in an effective transition state geometry which looks more product-like in the respective mode: Excitation in the umbrella bending mode results in a smaller, more product-like umbrella angle, and excitation in the C─H stretching mode results in a larger C─H distance. The extent of this effect can be directly related to the increased vibrational amplitudes as illustrated in Fig. 3B, where a contour plot of the potential energy surface, the separating ridge, and the effective transition state geometries are displayed. We connect the shifts in the effective transition state geometry to the vibrational excitations of the reactants using colored arrows. The size of an arrow indicates the increase of the vibrational amplitude resulting from one quantum of vibrational energy in the stretching and umbrella bending modes (see the Supplementary Materials for a more detailed description). These sizes agree well with the shifts of the effective transition state geometries.

The quantum state–resolved picture of the chemical reaction process emerging from the above analysis is a natural extension of textbook principles routinely applied in synthetic chemistry; in a kinetically controlled chemical reaction, the transition state plays the key role. Control of the reaction path can be achieved via designed modifications of the transition state, e.g., by altering substituents in the reacting molecules to drive the reaction in the desired direction. On the quantum state–resolved level, vibrational control of the reaction pathway works analogously. Depositing vibrational energy in a specific mode alters the geometry of the effective dynamical transition state. The geometry is no longer simply defined by the saddle point of the potential energy surface but shows a more product-like geometry in the vibrationally excited mode. The dynamics after passing the transition state and the product state distribution are determined by the geometry of the effective transition state.

This picture of vibrational control is unexpectedly simple and intuitive. However, it has to be noted that in chemistry, there is no rule without exception. In the title reaction, Fermi resonance–type state mixing between the single C─H stretch excited and the triple CD_3_ umbrella bending excited state results in identical product distributions for both states. Thus, while intuitive pictures can provide important guiding principles, experiment or predictive theory is indispensable to correctly describe the complex reality.

## MATERIALS AND METHODS

The present calculations use the quantum transition state concept (*39*–*42*) and the multiconfigurational time-dependent Hartree (MCTDH) approach (*43*–*46*) to obtain accurate state-to-state reaction probabilities. The reactive scattering event is decomposed into two half collisions describing the decay of the activated complex into reactants and products. Different coordinate systems are used in the reactant and product channels. A coordinate transformation of the eigenstates of the thermal flux operator links the two separate calculations. Generalized flux correlation functions (*42*) are used to compute the state-to-state reaction probabilities for vanishing total angular momentum. The 12-dimensional wave packet dynamics calculations are facilitated by the multilayer state-averaged MCTDH approach (*43*–*47*) and the correlation discrete variable representation (*46*, *48*, *49*). The ab initio potential energy surface developed by Xu *et al*. (*50*) is used. At the total energies considered, the reaction can be accurately described within the Born-Oppenheimer approximation, and detailed calculations for the H + CH_4_ → H_2_ + CH_3_ reaction (*51*) had already confirmed the essentially quantitative accuracy of the potential energy surface used. The description of the reactant channel used results of previous initial state-selected calculations (*35*). Further details regarding the numerical calculations and a description of the convergence tests are given in the Supplementary Materials. It should be noted that due to the higher mass of deuterium compared to hydrogen, much larger basis sets than in previous work on the H + CH_4_ → H_2_ + CH_3_ reaction (*8*) had to be used to achieve converged results.

The accurately computed reaction probabilities are interpreted using a simple model that uses the sudden approximation for the decay of the activated complex, a harmonic model of the reactant and product vibrations, and Gaussian-shaped transition state wave packets. A detailed description of the model and a complete list of all parameters used are given in the Supplementary Materials.

## Supplementary Materials

### This PDF file includes:

Supporting Text Figs. S1 to S21 Tables S1 and S2 References
