# Supplementary material for: Vibrational control of the reaction pathway in the H + CHD3 → H2 + CD3 reaction
Source: Sci Adv. 2022 Mar 30;8(13):eabm9820. doi: 10.1126/sciadv.abm9820 (PMC8967217; doi:10.1126/sciadv.abm9820)
Supplement: Supplementary file 1 — Supporting Text Figs. S1 to S21 Tables S1 and S2 References [file sciadv.abm9820_sm.pdf]

Science Advances
AAAS

Supplementary Materials for

Vibrational control of the reaction pathway in the $H + CHD_3 \rightarrow H_2 + CD_3$ reaction

Roman Ellerbrock, Bin Zhao, Uwe Manthe*

*Corresponding author. Email: uwe.manthe@uni-bielefeld.de

Published 30 March 2022, *Sci. Adv.* **8**, eabm9820 (2022)
DOI: 10.1126/sciadv.abm9820

The PDF file includes:

Supporting Text
Figs. S1 to S21
Tables S1 and S2
Legends for movies S1 to S3
References

Other Supplementary Material for this manuscript includes the following:

Movies S1 to S3

Supporting Text

Numerical Details

The present calculations employ the same methods used in previous full-dimensional calculations of initial-state-selected and state-to-states reaction probabilities for the $\mathrm{H}+\mathrm{CH}_4 \rightarrow \mathrm{H}_2+\mathrm{CH}_3$ *(19,20,51–54)* and $\mathrm{H}+\mathrm{CHD}_3 \rightarrow \mathrm{H}_2+\mathrm{CD}_3$ *(35–37)* reactions. In the following, we summarize the employed methods.

State-to-state reaction probabilities for vanishing total angular momentum J are obtained from combining independent calculations on reactant and product side via generalized flux correlation functions *(42,55)*. First, eigenstates of the thermal flux operator are computed. These eigenstates are propagated into the reactant and product asymptotic region. Initial and product state-selected reaction probabilities are obtained using flux correlation functions which include a projection onto the ro-vibrational eigenstates of the CHD_3 or CD_3 and H_2 fragments, respectively. State-to-state reaction probabilities are obtained by combining flux correlation matrix elements from reactant and product side.

The present calculations use different internal coordinate systems for the reactant and product sides. The curvilinear coordinate systems and kinetic energy operators used in the present calculations are taken from Refs. *(52)* and *(56)*. On the reactant side, the coordinate system is based on a H-(H-CD_3) Jacobi construction. The Jacobi vectors describing the positions of the two H atoms relative to the CD_3-fragment are parametrized by stereographic coordinates. The resulting coordinates for the H-atom in the CHD_3 reactant and the incoming H-atom are denoted by r, s, t and R, S, T, respectively. On the product side, the same parametrization is used but the scheme for the Jacobi-construction is (H-H)-(CD_3) where r, s, t are used to parametrize the H_2 fragment's Jacobi vector, and R,S,T are used to parametrized the vector pointing from H_2 to CD_3. The coordinate system for the methyl fragment is taken from Ref. *(56)*. The umbrella motion corresponds to θ and the coordinates φ and χ describe the *e*-symmetric H-bending motion. The coordinates ρ and $\vartheta_\rho, \phi_\rho$ describing the totally symmetric and *e*-symmetric stretching motions, are obtained from a spherical parametrization of the norms of the three Radau vectors. The body fixed coordinate system is tied to the methyl fragment. The trisector of the three Radau vectors defines the z-axis and one of the Radau vectors is fixed in the x-z-plane. All distance-type coordinates are mass weighted appropriately.

The quantum dynamics calculations are performed using the multilayer MCTDH approach *(43-46)*. The multilayer correlation discrete variable representation (CDVR) *(46,48,49)* approach is employed to compute potential integrals. The design of the multilayer MCTDH wavefunction representation closely follows previous work. Fig. S1 shows the wavefunction representation used in the thermal flux eigenstate calculations and the real-time propagations (see Ref. *(46)* for a detailed explanation of the diagrammatic notation). The number of single-particle functions (SPFs) used in the thermal flux eigenstate calculations and the real-time propagations are listed in Tab. S1; primitive basis or grid information is listed in Tab. S2. The present work uses converged

simulations of the reactant side that has been published elsewhere *(35)*. Here, we present results obtained from three different basis set combinations: $(s_{flux}, s_{reac}, s_{prod})$, $(A_{flux}, A_{reac}, A_{prod})$ and $(A_{flux}, A_{reac}, B_{prod})$. We distinguish the sets via the name of the product basis (s_{prod}, A_{prod}, or B_{prod}). The time-independent basis sets used in all calculations are listed in Tab. S2. The reference temperature used in the thermal flux operator was T = 2000 K. The thermal flux eigenstates were propagated for 150 fs on the reactant side and 105 fs on the product side. The dividing surface in the transition-state region is placed at r = 120 a.u. while the dividing surface in the product asymptotic area was set to R = 500 a.u.. A quartic absorbing potential starting at r = 145 a.u. and a maximum strength of $-i \cdot 10.368$ eV was used to decouple the reactant channel. Another absorbing potential with the same maximum strength was placed in the reactant asymptotic region starting at R = *1585* a.u.

For the asymptotic projection, the first two vibrational states of H_2 and the ground state and first five umbrella excitations of CD_3 were calculated. The convergence of the states with respect to number of SPFs and time-independent basis functions was verified. The results on the product channel are accumulated over all relative rotations of H_2 and CD_3 that couple to vanishing total angular momentum.

Convergence

We compare results obtained with sets s_{prod}, A_{prod} and B_{prod} to test convergence of the results. s_{prod} is our reference basis and A_{prod} and B_{prod} test convergence in the coordinates (r, s, t) and (R, S, T), respectively. Furthermore, we started independent calculations with additional SPFs in the remaining coordinates but stopped them after a few ten fs, because the additional SPFs remained unoccupied. Convergence of thermal flux eigenstates and real-time propagation to the reactant channel was confirmed in earlier studies *(36,38)*.

Fig. S2-S21 display state-to-state reaction probabilities for different vibrational and rotational states of the reactant, CHD_3, and different product vibrational states of CD_3 that are discussed in the main work. We find that results obtained with different basis sets match precisely for all vibrational and rotational states. Results typically differ at around 5% (and often less than that) which is smaller than the 10% error reported for reactant basis *(35)*. Note that errors here include not only the error from the product side but also from thermal flux eigenstate calculation and real-time propagation to the reactant side for the reported basis sets.

Illustrative movies

Movies S1-S3 illustrate the calculations and results. Data corresponding to the density matrix obtained by averaging over all propagated wave packets (weighted according to the reference temperature of 2000 K) is displayed.

A trajectory-like view of the simulated reaction process is shown in Movie S1. Here the different atoms are located at the expectation values of coordinate values, i.e., at their mean position. The approach of the reactants is labeled using negative times. The positions

corresponding to the initial wave packets employed in the numerical calculations, i.e., the thermal flux eigenstates, are reached at time t=0. The outgoing motion of the reactants is labeled using positive time.

Movie S2 illustrates the propagation in the reactant channel in more detail. 1D probability densities along the H-(H-CD_3)-distance (R), the H-CD_3-distance (r), and the umbrella bending angle θ are plotted. Probability densities in r and θ corresponding to the different relevant eigenstates of methane are also shown. They illustrate typical vibration amplitudes and help to rationalize the contribution of different vibrational excited states to the overall reactivity.

Movie S3 analogously illustrates the propagation in the product channel. Here a transformation of the coordinate system is required before the start of the propagation. 1D probability densities along the (H-H)-CD_3-distance coordinate R', the H-H-distance coordinate r', and the umbrella bending angle θ are plotted. Probabilities densities in θ corresponding to the different relevant eigenstates of methyl are displayed for comparison. They help to rationalize the (averaged) product state distribution.

A Sudden Approximation Based Model

The accurately computed state-to-state reaction probabilities can be convincingly interpreted using the simple model which employs the sudden approximation and harmonic models for all vibrations. Thus, the C-H stretching motion in the CHD_3 reactant is described by the Hamiltonian (atomic units and thus $\hbar = 1$ are used throughout this work)

$$\widehat{H} = \frac{1}{2\mu_r}\frac{d^2}{dr^2} + \frac{1}{2}\mu_r\omega_r^2(r - r_0)^2$$

with the reduced mass

$$\mu_r = \frac{m_H \cdot (m_C + m_H + 3m_D)}{m_H + (m_C + m_H + 3m_D)}.$$

r_0 denotes the equilibrium distance, ω_r the vibrational frequency in harmonic approximation, and m_C, m_H, and m_D denote the masses of the carbon, hydrogen, and deuterium atoms, respectively. Following the reduced-dimensional description developed by Palma and Clary *(57)*, the motion in the umbrella bending vibration in the CHD_3 reactant and CD_3 product is described in harmonic approximation by the Hamiltonian

$$\widehat{H} = \frac{1}{2\mu_\theta r_{CD}^2}\frac{d^2}{d\theta^2} + \frac{1}{2}\mu_\theta r_{CD}^2\omega_\theta^2(\theta - \theta_0)^2$$

where the reduced mass is defined by

$$\frac{1}{\mu} = \sin^2\theta_0 \cdot \frac{3m_D m_C}{3m_D + m_C} + \cos^2\theta_0 \cdot \frac{1}{m_D},$$

θ_0 and r_{CD} are the equilibrium values of the umbrella bending angle and the C-D distance, and ω_θ is the vibrational frequency in harmonic approximation. Note that the equilibrium values θ_0 and vibrational frequencies ω_θ differ significantly for the CHD_3 reactant and CD_3 product.

Within the sudden approximation, transition probabilities are computed by directly mapping an initial wavefunction ϕ onto a set of final product states ψ_ν, i.e., the transition probabilities w_ν are given by the Franck-Condon factors $w_\nu = |\langle\phi|\psi_\nu\rangle|^2$. The simplest model employs harmonic oscillator product states and an initial state given by a Gaussian wavefunction corresponding to the ground state of a displaced harmonic oscillator with same frequency. Then the transition probabilities for an oscillator with mass m and frequency ω are given by

$$w_\nu = \frac{1}{\nu!} S^\nu e^{-S}, S = \frac{m\omega}{2}\Delta x^2$$

where S is the Huang-Rhys-Parameter and Δx the displacement of the center of φ relative to the equilibrium geometry of the harmonic oscillator. This simple model has been used to obtain the description presented in Fig. 2. The product state distributions presented in Fig.1 can be associated with Huang-Rhys parameters for the umbrella bending mode in the CD_3 product. Reaction from the ground vibrational state of CHD_3 shows a product distribution which peaks at a quantum number $\nu = 1$ and shows approximately equal probabilites for the $\nu = 0$ and $\nu = 2$ product states. This product distribution corresponds to a Huang-Rhys parameter $S = \sqrt{2}$. Reaction from the umbrella bending excited state of CHD_3 ($\nu_{umbrella}(\mathrm{CHD_3}) = 1$) shows a product distribution with approximately equal probabilites for the $\nu = 0$ and $\nu = 1$ product states which corresponds to a Huang-Rhys-parameter $S = 1$. Reaction from the C-H stretch excited state of CHD3 ($\nu_{stretch}(\mathrm{CHD_3}) = 1$) shows a product distribution with approximately equal probabilites for the $\nu = 1$ and $\nu = 2$ product states corresponding to a Huang-Rhys parameter $S = 2$. Based on these values of the Huang-Rhys parameter, the transition state wavepackets which give rise to these product state distributions can be reconstructed. Using $\omega = 444\mathrm{cm}^{-1}$, $\theta_0 = 90°$, and $r_{CD} =$ 1.0777Å, the displacements $\Delta\theta$ of 12.3°, 10.3° , and 14.6° corresponding to the Huang-Rhys-Parameters S of $\sqrt{2}$, 1, and 2, respectively, are obtained. These values are employed in Figs. 2 and 3. The vibrational amplitudes in the CHD_3 reactant are calculated using a simple semiclassical picture: the probability density of a vibrational wavefunctions is large only in the classically region. Thus, we equate the amplitude A_ν of a vibration in the quantum state ν with the distance between the equilibrium goemetry and the classical turning point. For a harmonic oscillator in state ν with an available energy the amplitude is thus calculated via

$$E_\nu = V(A_\nu) \Longrightarrow \omega(\nu + 0.5) = \frac{1}{2} m\omega^2 A_\nu^2.$$

The increase in the vibrational amplitude resulting from one quantum of excitation, $\Delta A = A_1 - A_0$, defines the size of the arrows displayed in Fig.3b. Using $\omega_\theta = 1009\mathrm{cm}^{-1}$, $\omega_r = 2990\mathrm{cm}^{-1}$, $\theta = 109.47°$, and $r_{CD} = 1.0777$Å or the CHD_3 molecule, ΔA values of 1.8° and 0.076 Å are obtained for the umbrella bending and C-H stretching modes, respectively.

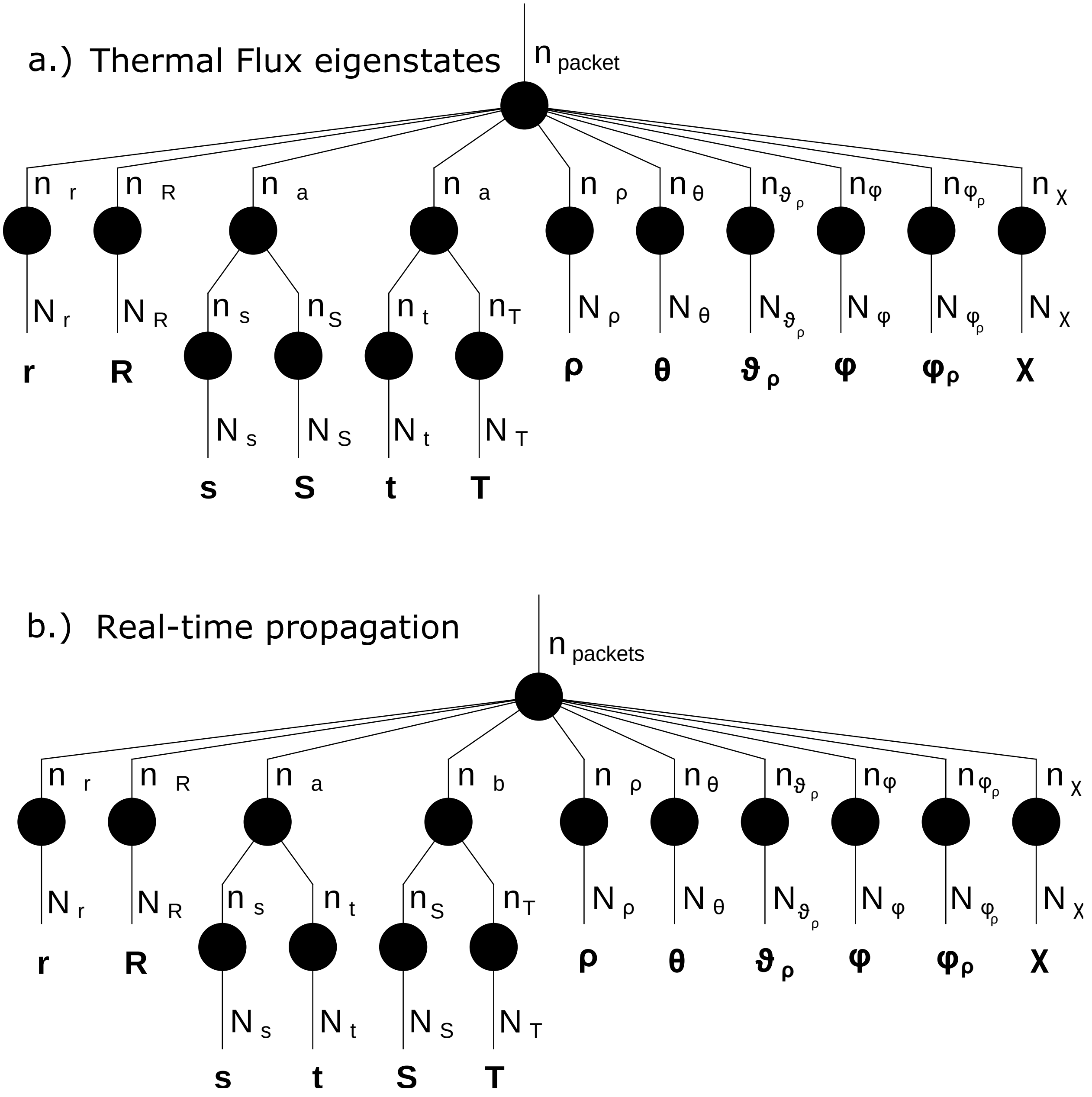

Fig. S1. MCTDH wavefunction representation. Diagrammatic representations of the multilayer MCTDH wavefunction representations used in thermal flux eigenstate calculations and real-time propagations.

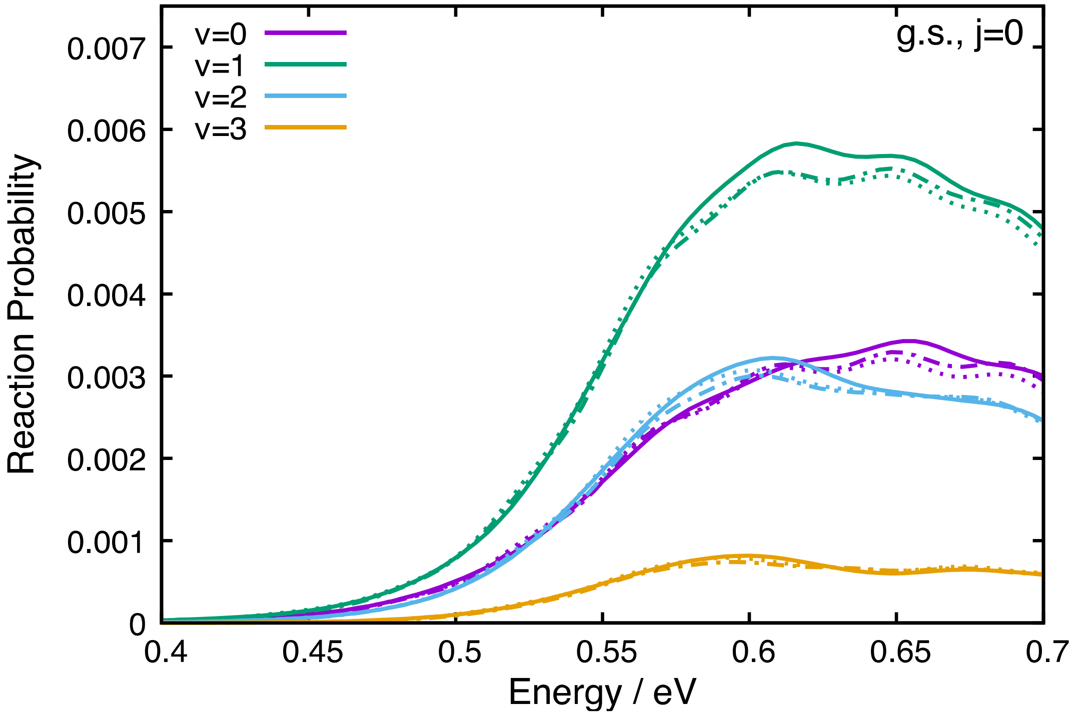

Fig. S2. State-to-state reaction probabilities. Probabilities for reaction starting in the vibrational ground state of CHD_3 (j=0) and ending in different product vibrational states of CD_3. Solid, dashed, and dotted lines correspond to results obtained with basis s, A and B, respectively.

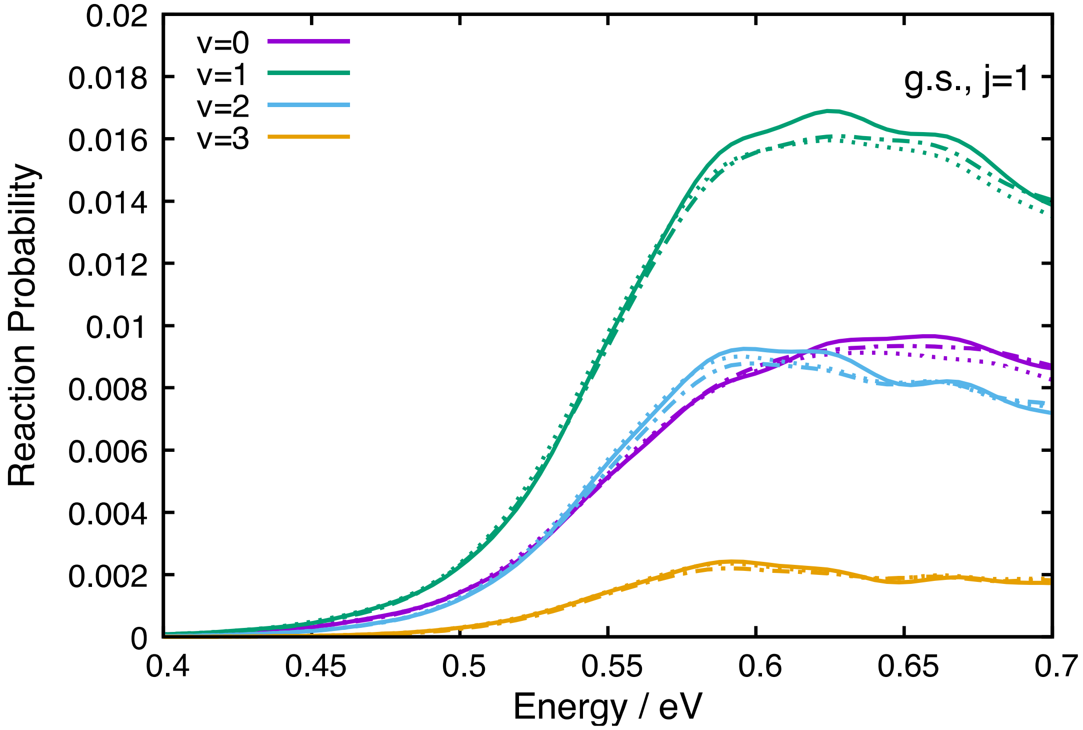

Fig. S3. State-to-state reaction probabilities. Probability for reaction starting in the vibrational ground state of CHD_3 (j=1) and ending in different product vibrational states of CD_3. Solid, dashed, and dotted lines correspond to results obtained with basis s, A and B, respectively.

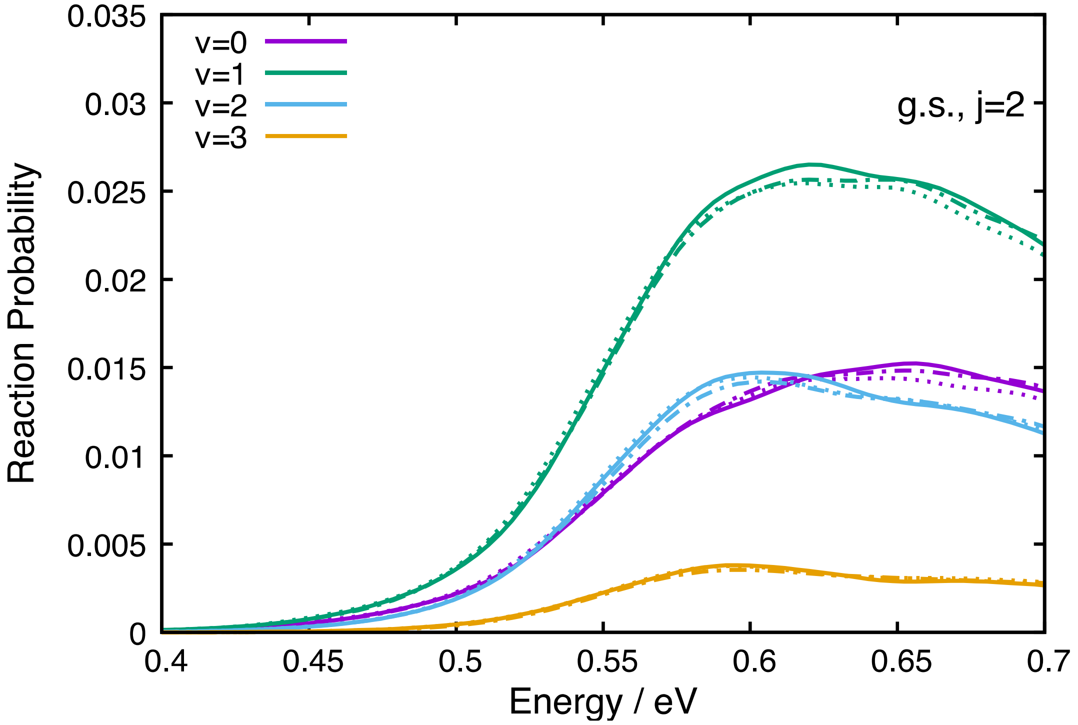

Fig S4. State-to-state reaction probabilities. Probability for reaction starting in the vibrational ground state of CHD_3 (j=2) and ending in different product vibrational states of CD_3. Solid, dashed, and dotted lines correspond to results obtained with basis s, A and B, respectively.

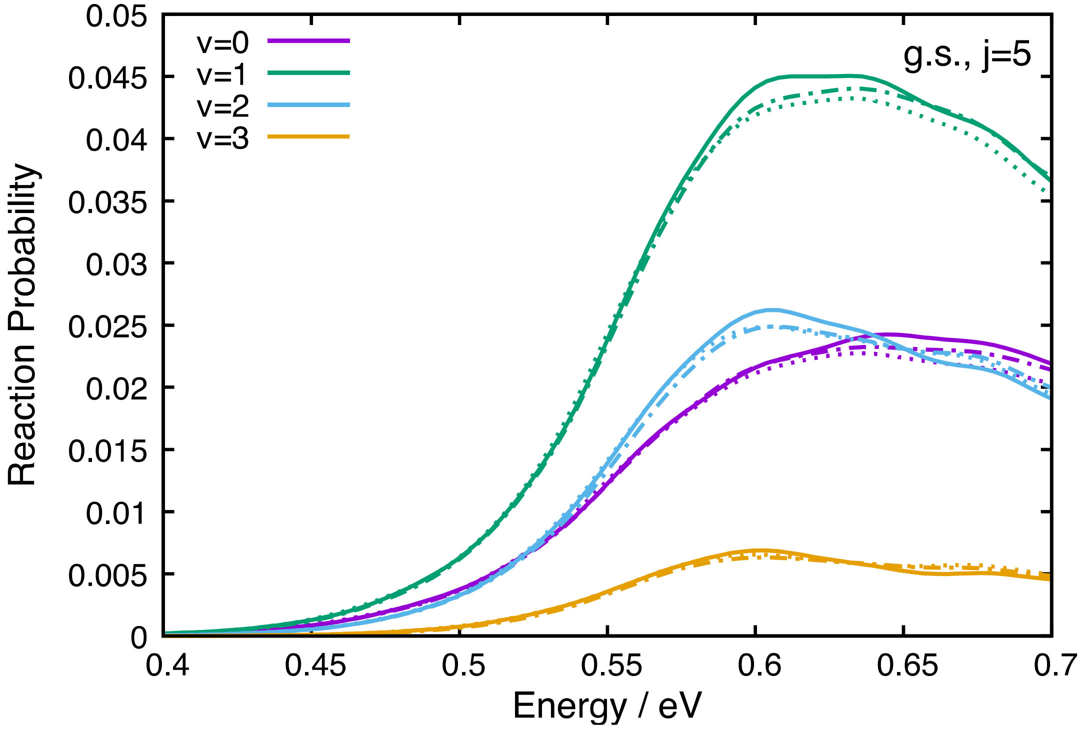

Fig S5. State-to-state reaction probabilities. Probability for reaction starting in the vibrational ground state of CHD_3 (j=5) and ending in different product vibrational states of CD_3. Solid, dashed, and dotted lines correspond to results obtained with basis s, A and B, respectively.

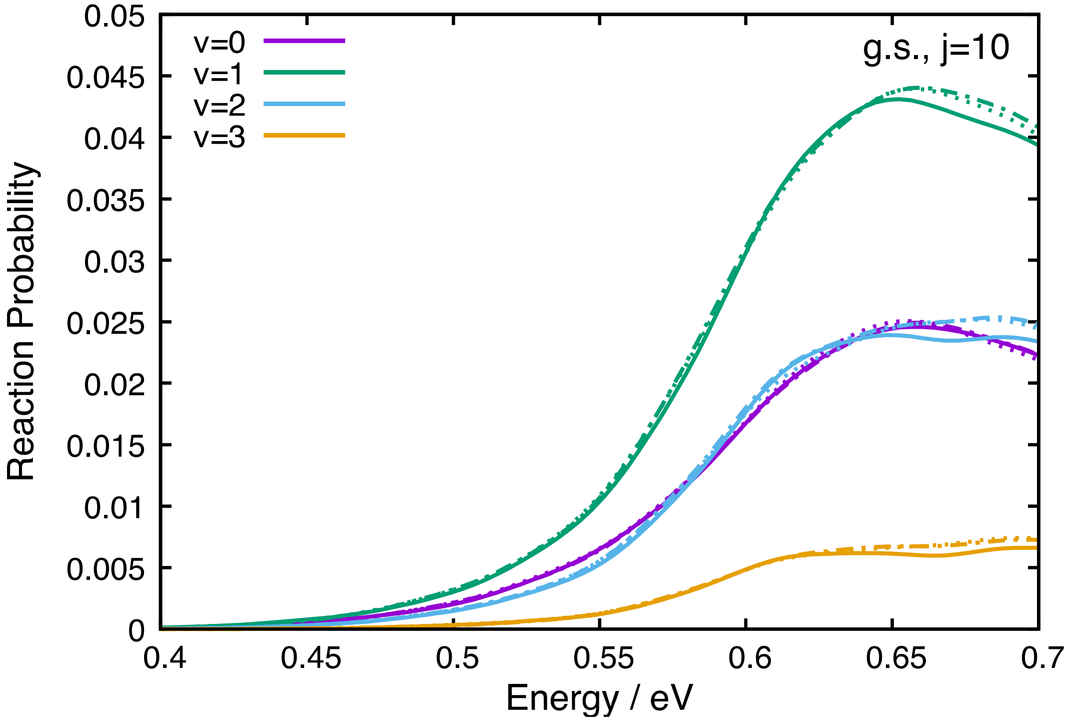

Fig S6. State-to-state reaction probabilities. Probability for reaction starting in the vibrational ground state of CHD_3 (j=10) and ending in different product vibrational states of CD_3. Solid, dashed, and dotted lines correspond to results obtained with basis s, A and B, respectively.

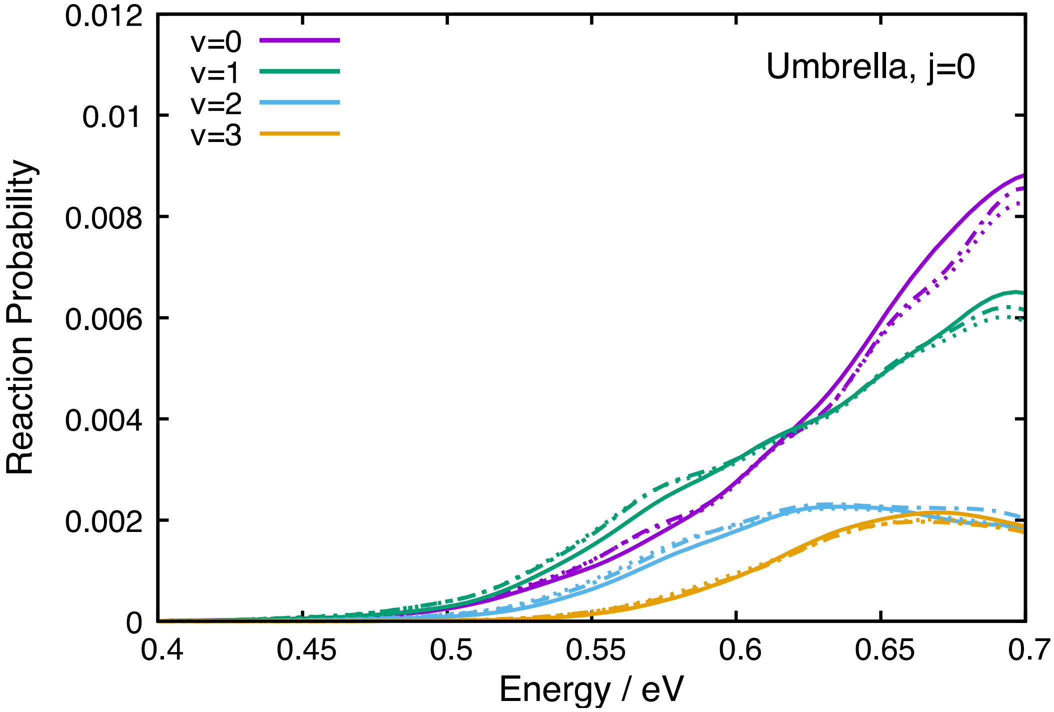

Fig S7. State-to-state reaction probabilities. Probability for reaction starting in the umbrella excited state of CHD_3 (j=0) and ending in different product vibrational states of CD_3. Solid, dashed, and dotted lines correspond to results obtained with basis s, A and B, respectively.

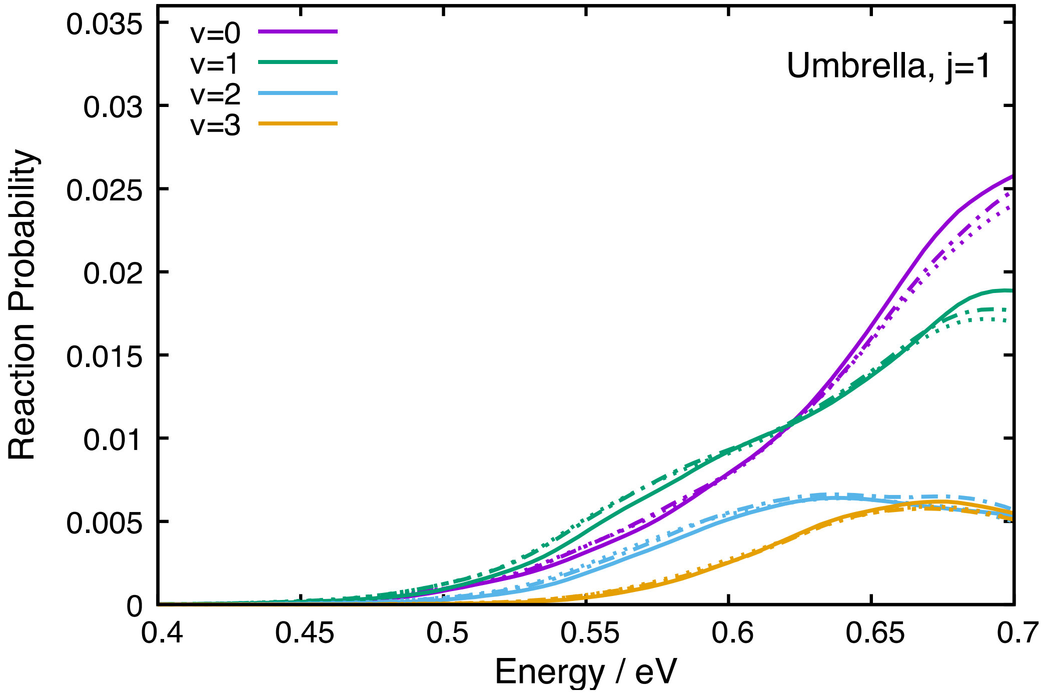

Fig. S8. State-to-state reaction probabilities. Probability for reaction starting in the umbrella excited state of CHD_3 (j=1) and ending in different product vibrational states of CD_3. Solid, dashed, and dotted lines correspond to results obtained with basis s, A and B, respectively.

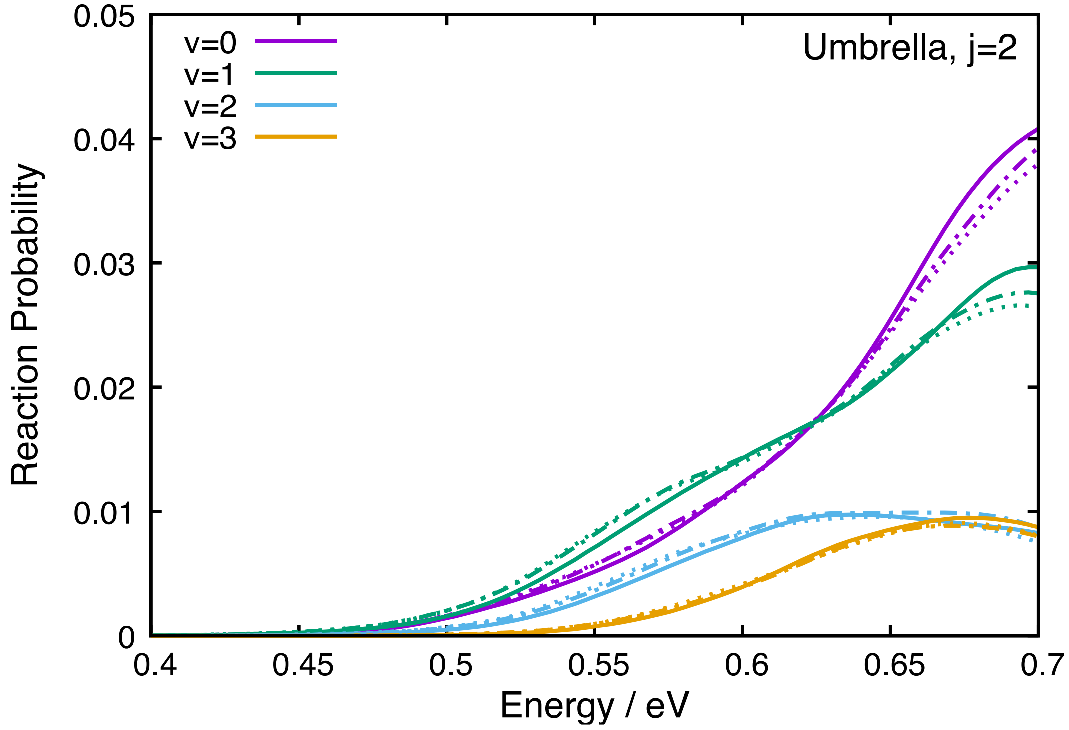

Fig. S9. State-to-state reaction probabilities. Probability for reactions starting in the umbrella excited state of CHD_3 (j=2) and ending in different product vibrational states of CD_3. Solid, dashed, and dotted lines correspond to results obtained with basis s, A and B, respectively.

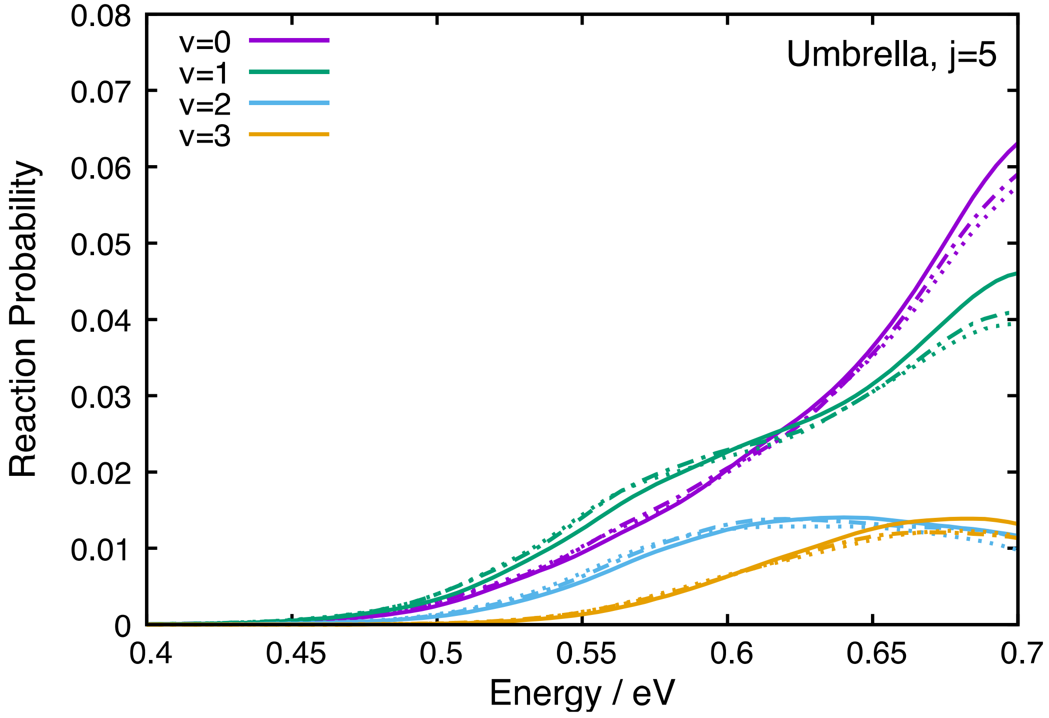

Fig. S10. State-to-state reaction probabilities. Probability for reaction starting in the umbrella excited state of CHD_3 (j=5) and ending in different product vibrational states of CD_3. Solid, dashed, and dotted lines correspond to results obtained with basis s, A and B, respectively.

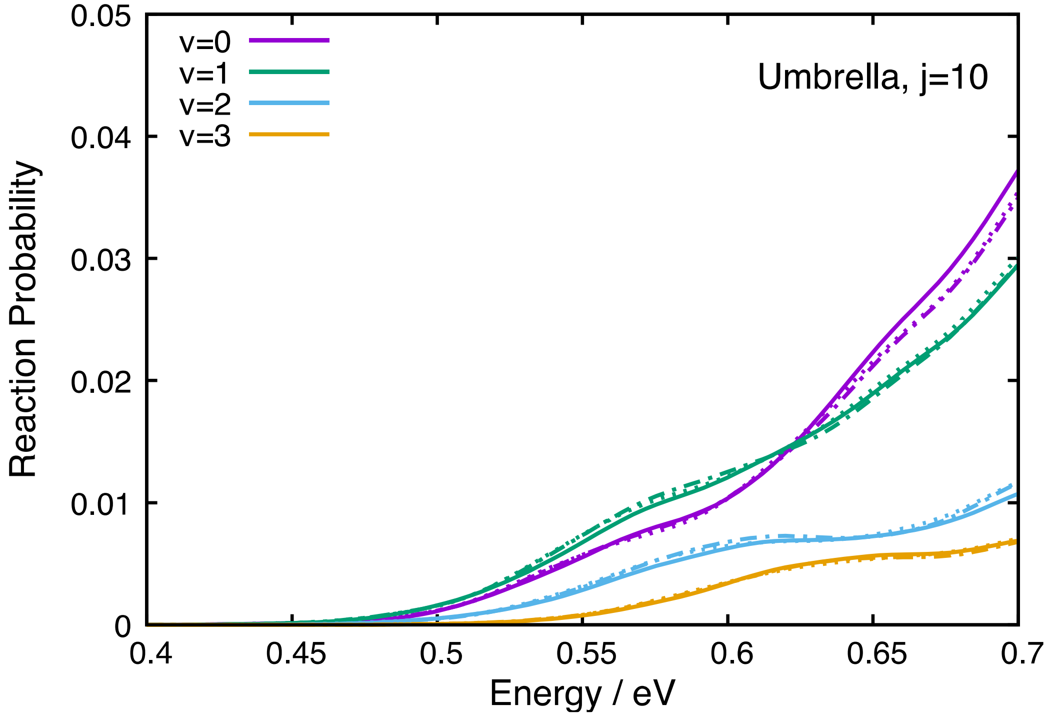

Fig. S11. State-to-state reaction probabilities. Probability for reaction starting in the umbrella excited state of CHD_3 (j=10) and ending in different product vibrational states of CD_3. Solid, dashed, and dotted lines correspond to results obtained with basis s, A and B, respectively.

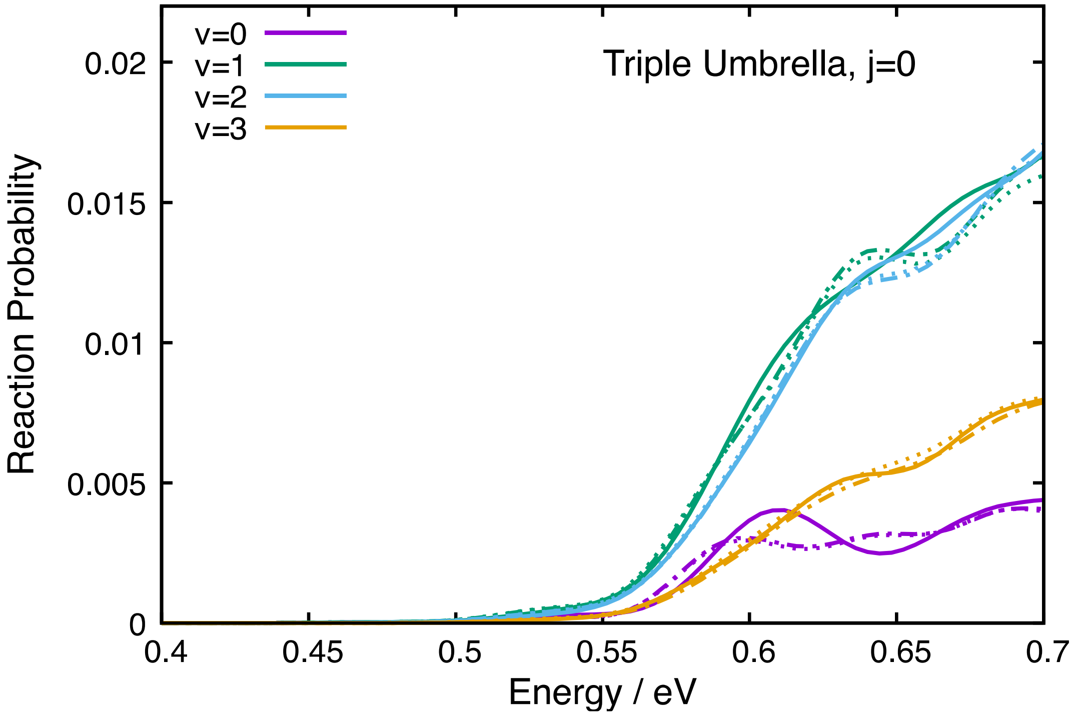

Fig. S12. State-to-state reaction probabilities. Probability for reaction starting in the triple umbrella excited state of CHD_3 (j=0) and ending in different product vibrational states of CD_3. Solid, dashed, and dotted lines correspond to results obtained with basis s, A and B, respectively.

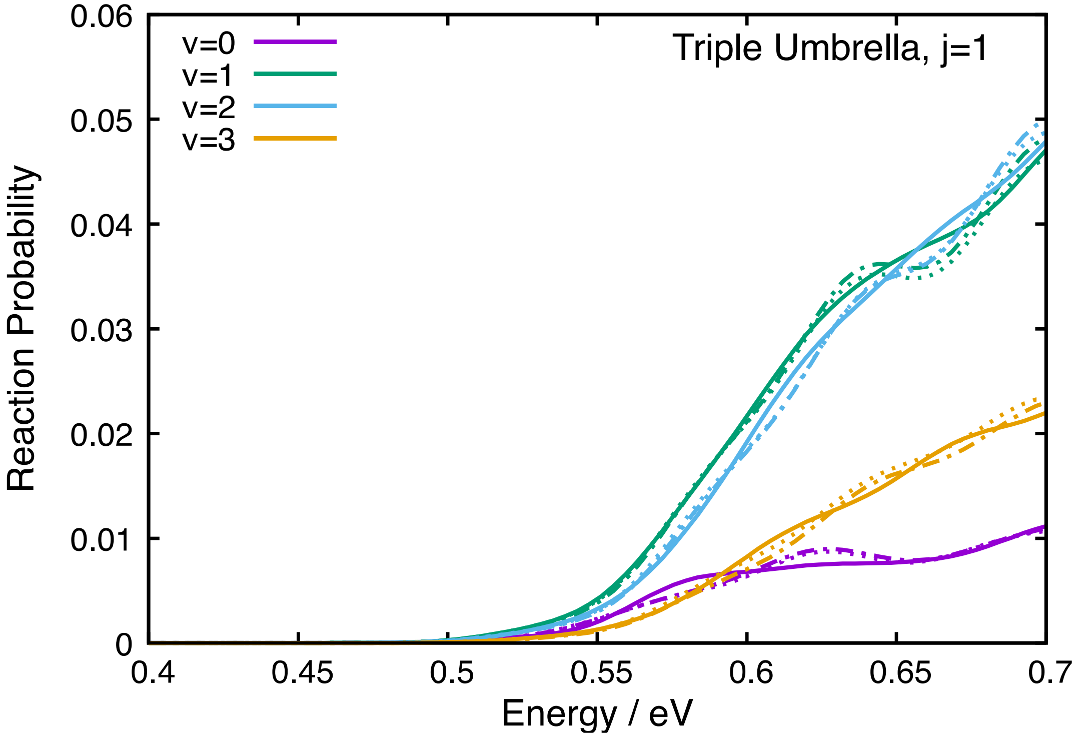

Fig. S13. State-to-state reaction probabilities. Probability for reaction starting in the triple umbrella excited state of CHD_3 (j=1) and ending in different product vibrational states of CD_3. Solid, dashed, and dotted lines correspond to results obtained with basis s, A and B, respectively.

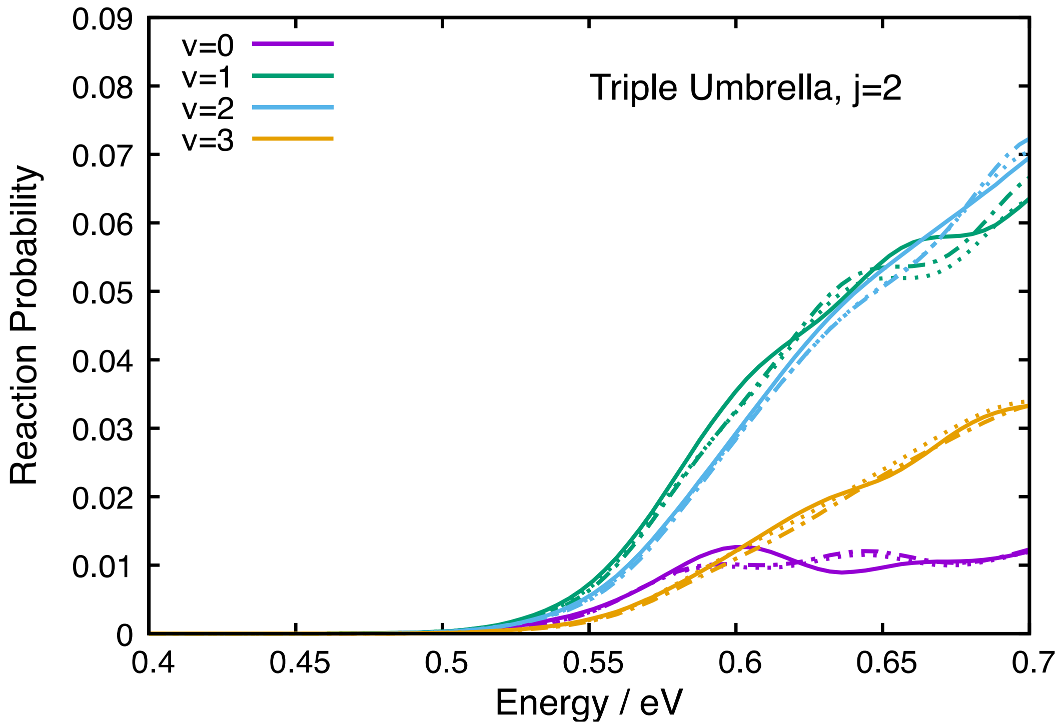

Fig. S14. State-to-state reaction probabilities. Probability for reaction starting in the triple umbrella excited state of CHD_3 (j=2) and ending in different product vibrational states of CD_3. Solid, dashed, and dotted lines correspond to results obtained with basis s, A and B, respectively.

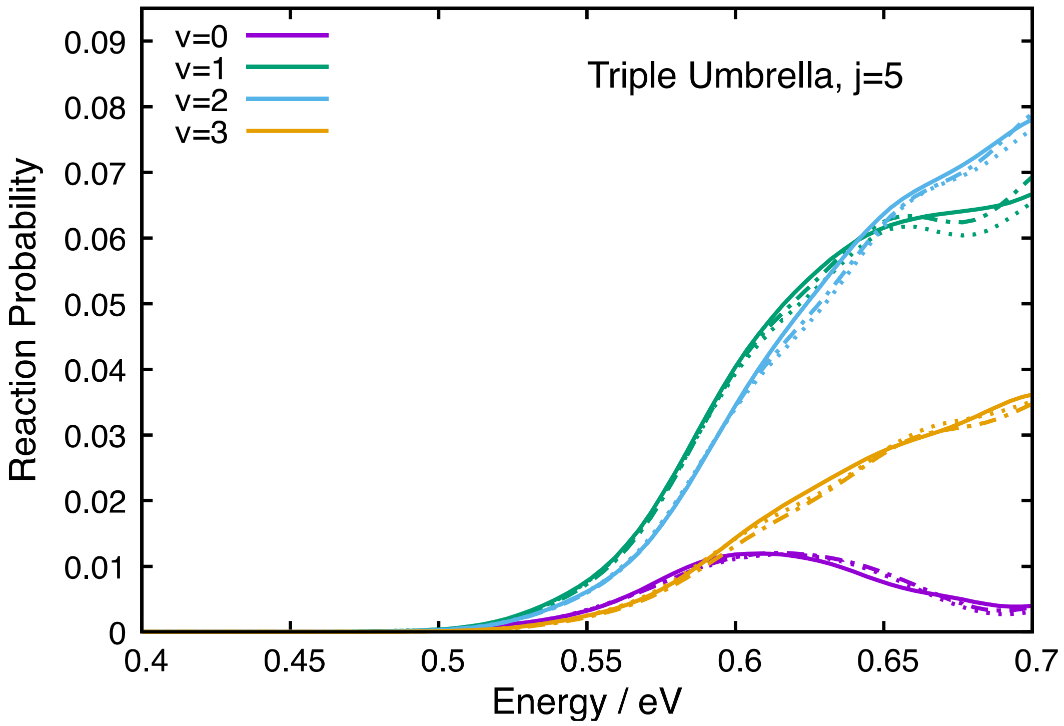

Fig. S15. State-to-state reaction probabilities. Probability for reaction starting in the triple umbrella excited state of CHD_3 (j=5) and ending in different product vibrational states of CD_3. Solid, dashed, and dotted lines correspond to results obtained with basis s, A and B, respectively.

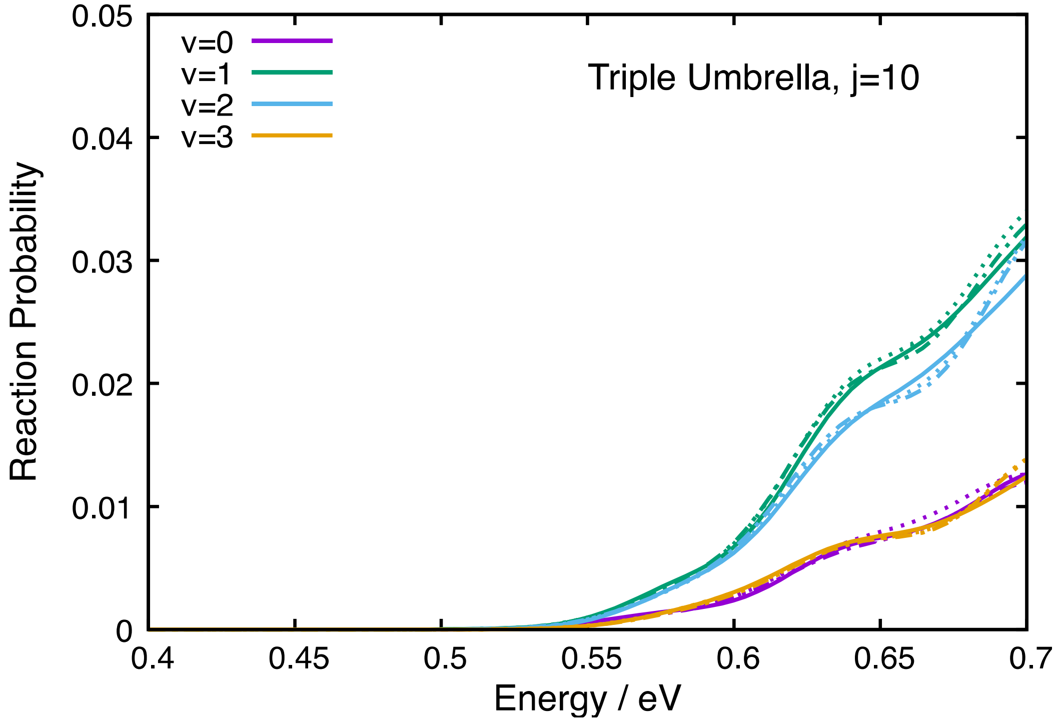

Fig. S16. State-to-state reaction probabilities. Probability for reaction starting in the triple umbrella excited state of CHD_3 (j=10) and ending in different product vibrational states of CD_3. Solid, dashed, and dotted lines correspond to results obtained with basis s, A and B, respectively.

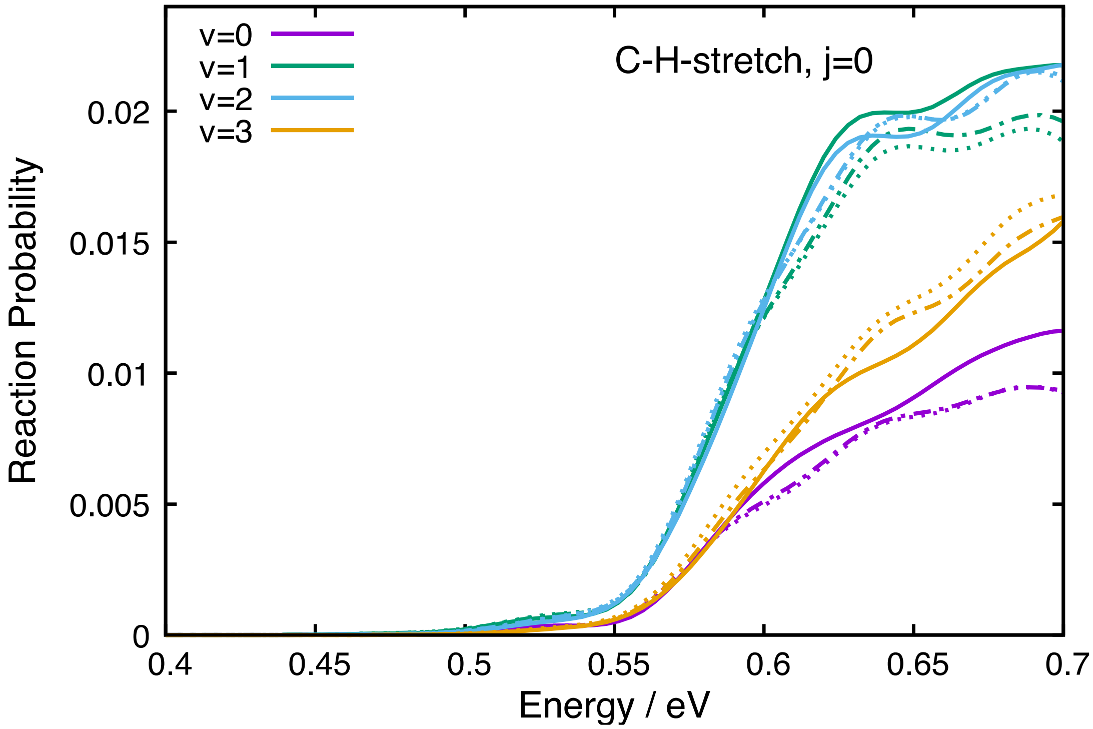

Fig. S17. State-to-state reaction probabilities. Probability for reaction starting in the C-H-stretch excited state of CHD_3 (j=0) and ending in different product vibrational states of CD_3. Solid, dashed, and dotted lines correspond to results obtained with basis s, A and B, respectively.

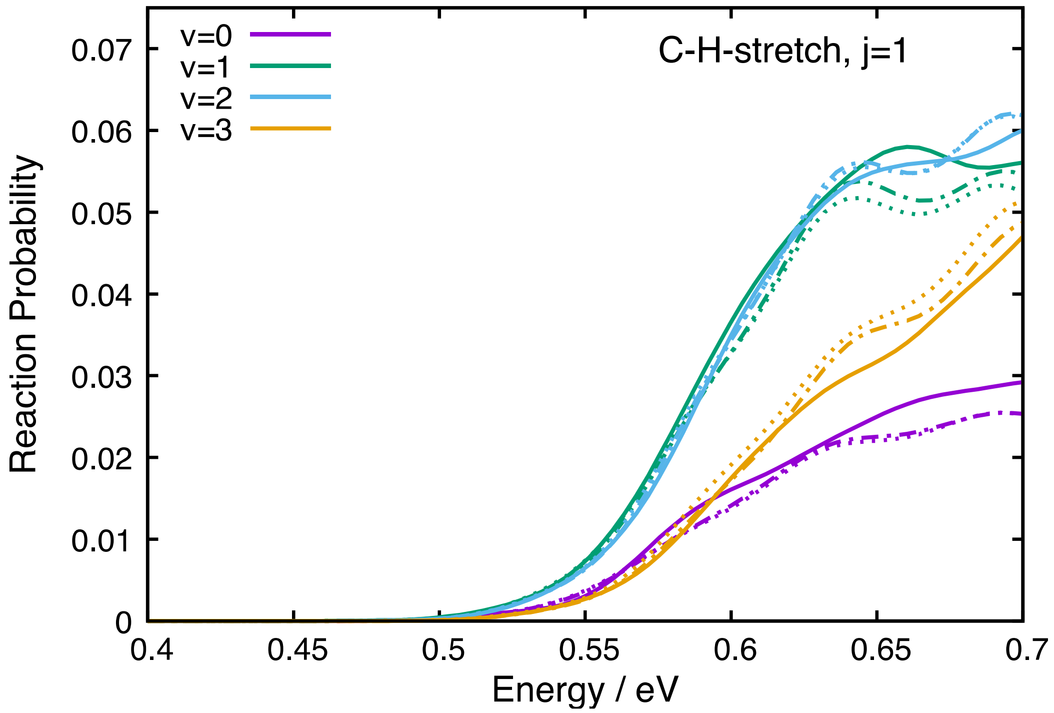

Fig. S18. State-to-state reaction probabilities. Probability for reaction starting in the C-H-stretch excited state of CHD_3 (j=1) and ending in different product vibrational states of CD_3. Solid, dashed, and dotted lines correspond to results obtained with basis s, A and B, respectively.

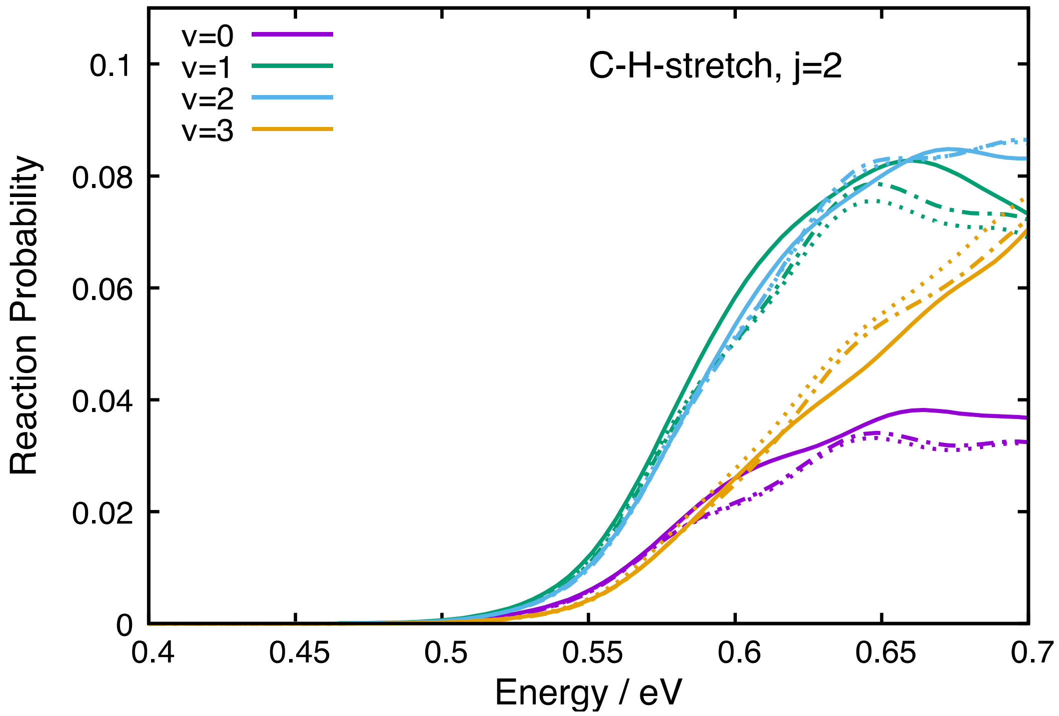

Fig. S19. State-to-state reaction probabilities. Probability for reaction starting in the C-H-stretch excited state of CHD_3 (j=2) and ending in different product vibrational states of CD_3. Solid, dashed, and dotted lines correspond to results obtained with basis s, A and B, respectively.

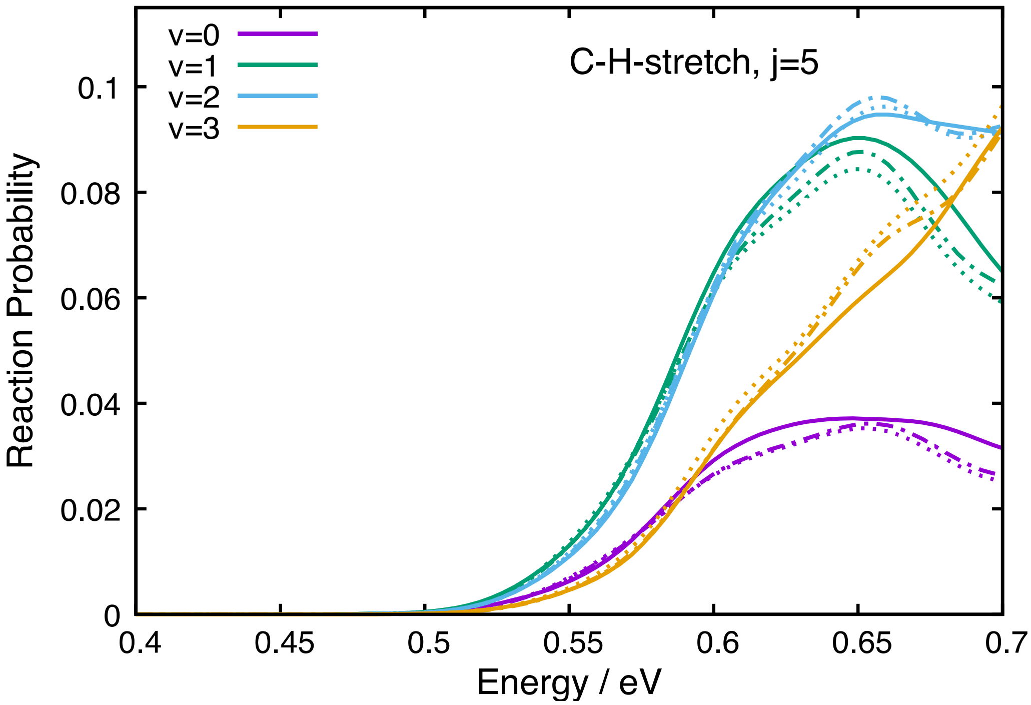

Fig. S20. State-to-state reaction probabilities. Probability for reaction starting in the C-H-stretch excited state of CHD_3 (j=5) and ending in different product vibrational states of CD_3. Solid, dashed, and dotted lines correspond to results obtained with basis s, A and B, respectively.

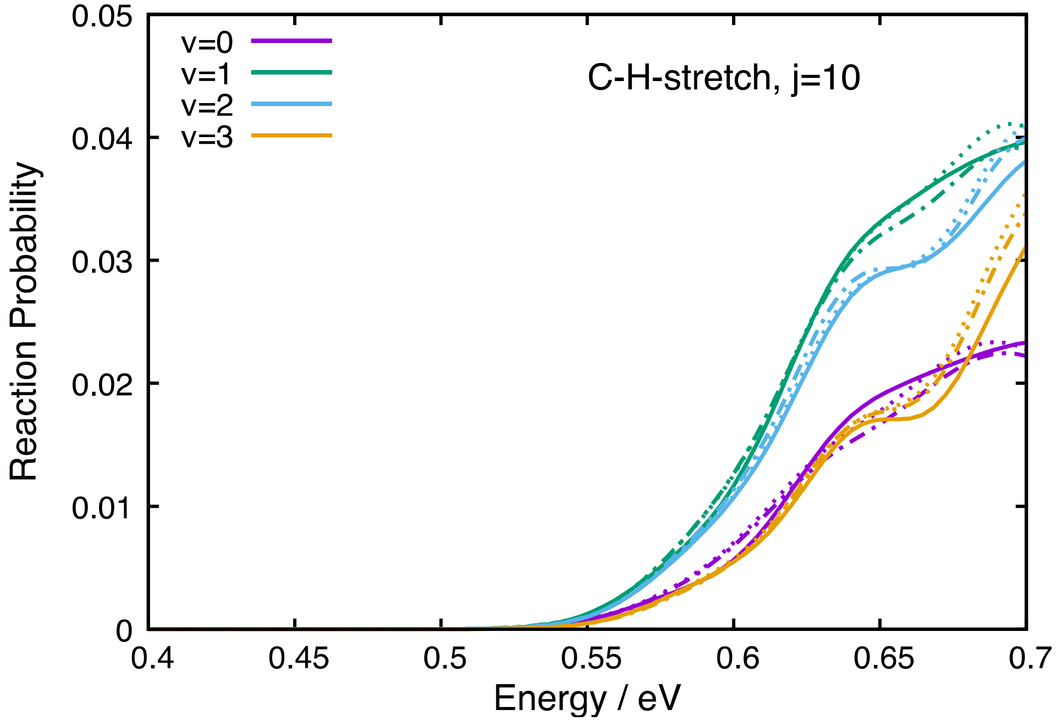

Fig. S21. State-to-state reaction probabilities. Probability for reaction starting in the C-H-stretch excited state of CHD_3 (j=10) and ending in different product vibrational states of CD_3. Solid, dashed, and dotted lines correspond to results obtained with basis s, A and B, respectively.

	Thermal flux		Reactants		Products		
	S$_{flux}$	A$_{flux}$	S$_{reac}$	A$_{reac}$	S$_{prod}$	A$_{prod}$	B$_{prod}$
n_r	24	24	24	24	24	24	24
n_r	6	8	7	9	6	6	7
n_R	6	8	9	9	13	13	15
n_a	11	14	15	21	15	21	15
n_s, n_t	7	9	5	6	8	10	8
n_b	11	14	9	9	15	21	15
n_S, n_T	9	11	8	8	8	10	8
n_θ	5	7	8	8	9	9	11
$n_\rho, n_{\vartheta_\rho}, n_{\phi_\rho}, n_\varphi, n_\chi$	1	1	1	1	1	1	1

Table S1. Single-particle function basis sets. Numbers of single-particle functions in the multi-layer representation of the MCTDH wave functions used in real-time propagations on the product side.

	Type	Size	Range
N_r	FFT	32	(10.0, 186.0)
N_R	FFT	464	(10.5, 1650.0)
N_s, N_t	FFT	160	(-7.0, 7.0)
N_S, N_T	FFT	384	(-7.0, 7.0)
N_θ	Hermite-DVR	64	
N_ρ	Hermite-DVR	10	
$N_{\vartheta_\rho}, N_{\phi_\rho}$	Hermite-DVR	8	
N_φ, N_χ	Hermite-DVR	16	

Table S2. Time-independent basis set representations. Sizes and types of the time-independent (primitive) basis sets used to represent the bottom layer single-particle functions.

Movie Captions

Movie S1. Trajectory-like view of the simulated reaction process. The motion of the mean position of the atoms in course of the reaction process is illustrated. The approach of the reactants is labeled using negative times while the outgoing motion of the products is labeled by positive time (see text for more details).

Movie S2. Propagation in the reactant channel. 1D probability densities along the H-(H-CD3)-distance (R), the H-CD3-distance (r), and the umbrella bending angle θ are shown. Probability densities in r and θ corresponding to different relevant eigenstates of methane indicate typical vibrational amplitudes (see text for more details).

Movie S3. Propagation in the product channel. 1D probability densities along the (H-H)-CD3-distance (R'), the H-H-distance (r'), and the umbrella bending angle θ are shown. Probabilities densities in θ corresponding to different relevant eigenstates of methyl indicate typical vibrational amplitudes (see text for more details).

REFERENCES AND NOTES

1. S. Yan, Y.-T. Wu, B. Zhang, X.-F. Yue, K. Liu, Do vibrational excitations of CHD_3 preferentially promote reactivity toward the chlorine atom? *Science* **316**, 1723–1726 (2007).

2. S. Yan, Y.-T. Wu, B. Zhang, X.-F. Yue, K. Liu, Tracking the energy flow along the reaction path. *Proc. Natl. Acad. Sci. U.S.A.* **105**, 12667–12672 (2008).

3. F. Wang, J.-S. Lin, K. Liu, Steric control of the reaction of CH stretch-excited CHD_3 with chlorine atom. *Science* **331**, 900–903 (2011).

4. F. Wang, K. Liu, T. P. Rakitzis, Revealing the stereospecific chemistry of the reaction of Cl with aligned $CHD_3(\nu_1 = 1)$. *Nat. Chem.* **4**, 636–641 (2012).

5. J. Lin, J. Zhou, W. Shiu, K. Liu, State-specific correlation of coincident product pairs in the F + CD_4 reaction. *Science* **300**, 966–969 (2003).

6. W. Zhang, H. Kawamata, K. Liu, CH stretching excitation in the early barrier $\mathrm{F} + \mathrm{CHD}_3$ reaction inhibits CH bond cleavage. *Science* **325**, 303–306 (2009).

7. Z. Chen, J. Chen, R. Chen, T. Xie, X. Wang, S. Liu, G. Wu, D. Dai, X. Yang, D. H. Zhang, Reactivity oscillation in the heavy-light-heavy Cl + CH_4 reaction. *Proc. Natl. Acad. Sci. U.S.A.* **117**, 9202–9207 (2020).

8. R. Welsch, U. Manthe, Loss of memory in $\mathrm{H} + \mathrm{CH} - 4 \rightarrow \mathrm{H}_2 + \mathrm{CH}_3$ state-to-state reactive scattering. *J. Phys. Chem. Lett.* **6**, 338–342 (2015).

9. X. Yang, State-to-state dynamics of elementary bimolecular reactions. *Annu. Rev. Phys. Chem.* **58**, 433–459 (2007).

10. K. Liu, Recent advances in crossed-beam studies of bimolecular reactions. *J. Chem. Phys.* **125**, 132307 (2006).

11. B. Fu, X. Shan, D. H. Zhang, D. C. Clary, Recent advances in quantum scattering calculations on polyatomic bimolecular reactions. *Chem. Soc. Rev.* **46**, 7625–7649 (2017).

12. T. Wu, H.-J. Werner, U. Manthe, First-principles theory for the $\mathrm{H} + \mathrm{CH}_4 \rightarrow \mathrm{H}_2 + \mathrm{CH}_3$ reaction. *Science* **306**, 2227–2229 (2004).

13. L. Che, Z. Ren, X. Wang, W. Dong, D. Dai, X. Wang, D. H. Zhang, X. Yang, L. Sheng, G. Li, H.-J. Werner, F. Lique, M. H. Alexander, Breakdown of the born-oppenheimer approximation in the F+ *o*-D_2 → DF + D reaction. *Science* **317**, 1061–1064 (2007).

14. C. Xiao, X. Xu, S. Liu, T. Wang, W. Dong, T. Yang, Z. Sun, D. Dai, X. Xu, D. H. Zhang, X. Yang, Experimental and theoretical differential cross sections for a four-atom reaction: HD+OH → H_2O+D. *Science* **333**, 440–442 (2011).

15. M. Yang, D. H. Zhang, S.-Y. Lee, A seven-dimensional quantum study of the H+CH_4 reaction. *J. Chem. Phys.* **117**, 9539–9542 (2002).

16. W. Zhang, Y. Zhou, G. Wu, Y. Lu, H. Pan, B. Fu, Q. Shuai, L. Liu, S. Liu, L. Zhang, B. Jiang, D. Dai, S. Lee, Z. Xie, B. Braams, J. Bowman, M. Collins, D. Zhang, X. Yang, Depression of reactivity by the collision energy in the single barrier H + $CD_4 \rightarrow$ HD + CD_3 reaction. *Proc. Natl. Acad. Sci. U.S.A.* **107**, 12782–12785 (2010).

17. Y. Zhou, C. Wang, D. H. Zhang, Effects of reagent vibrational excitation on the dynamics of the $H + CHD_3 \rightarrow H_2 + CD_3$ reaction: A seven-dimensional time-dependent wave packet study. *J. Chem. Phys.* **135**, 024313 (2011).

18. Y. Wang, J. Li, L. Y. Chen, Y. P. Lu, M. H. Yang, D. H. Zhang, H. Guo, Mode specific dynamics of the H_2+CH_3→H+CH_4 reaction studied using quasi-classical trajectory and eight-dimensional quantum dynamics methods. *J. Chem. Phys.* **143**, 154307 (2015).

19. G. Schiffel, U. Manthe, Communications: A rigorous transition state based approach to state-specific reaction dynamics: Full-dimensional calculations for H+CH_4 → H_2+CH_3. *J. Chem. Phys.* **132**, 191101 (2010).

20. R. Welsch, U. Manthe, Communication: Ro-vibrational control of chemical reactivity in H+CH_4→ H_2+CH_3: Full-dimensional quantum dynamics calculations and a sudden model. *J. Chem. Phys.* **141**, 051102 (2014).

21. Z. Zhao, Z. Zhang, S. Liu, D. H. Zhang, Dynamical barrier and isotope effects in the simplest substitution reaction via walden inversion mechanism. *Nat. Commun.* **8**, 14506 (2017).

22. Z. Zhang, F. Gatti, D. H. Zhang, Full-dimensional quantum mechanical calculations of the reaction probability of the H + CH_4 reaction based on a mixed Jacobi and Radau description. *J. Chem. Phys.* **152**, 201101 (2020).

23. Z. Zhang, Y. Zhou, D. H. Zhang, G. Czako, J. M. Bowman, Theoretical study of the validity of the polanyi rules for the late-barrier Cl + CHD_3 reaction. *J. Phys. Chem. Lett.* **3**, 3416–3419 (2012).

24. N. Liu, M. Yang, An eight-dimensional quantum dynamics study of the Cl+CH_4→HCl+ CH_3 reaction. *J. Chem. Phys.* **143**, 134305 (2015).

25. M. Yang, S.-Y. Lee, D. H. Zhang, Seven-dimensional quantum dynamics study of the O (^3P)+CH_4 reaction. *J. Chem. Phys.* **126**, 064303 (2007).

26. R. Liu, M. Yang, G. Czako, J. M. Bowman, J. Li, H. Guo, Mode selectivity for a "Central" barrier reaction: Eight-dimensional quantum studies of the O(^3P) + CH_4→ OH + CH_3 reaction on an ab initio potential energy surface. *J. Phys. Chem. Lett.* **3**, 3776–3780 (2012).

27. J. Qi, H. Song, M. Yang, J. Palma, U. Manthe, H. Guo, Communication: Mode specific quantum dynamics of the F+CHD_3→HF+CD_3 reaction. *J. Chem. Phys.* **144**, 171101 (2016).

28. J. C. Polanyi, Some concepts in reaction dynamics. *Science* **236**, 680–690 (1987).

29. R. Liu, F. Wang, B. Jiang, G. Czako, M. Yang, K. Liu, H. Guo, Rotational mode specificity in the Cl + $CHD_3 \rightarrow$ HCl + CD_3 reaction. *J. Chem. Phys.* **141**, 074310 (2014).

30. B. Jiang, H. Guo, Relative efficacy of vibrational vs. translational excitation in promoting atom-diatom reactivity: Rigorous examination of Polanyi's rules and proposition of sudden vector projection (SVP) model. *J. Chem. Phys.* **138**, 234104 (2013).

31. B. Jiang, H. Guo, Mode specificity, bond selectivity, and product energy disposal in X+CH_4/CHD_3 (X=H, F, O(^{3}P), Cl, and OH) hydrogen abstraction reactions: Perspective from sudden vector projection model. *J. Chin. Chem. Soc.* **61**, 847–859 (2014).

32. B. Zhao, Z. Sun, H. Guo, State-to-state mode specificity: Energy sequestration and flow gated by transition state. *J. Am. Chem. Soc.* **137**, 15964–15970 (2015).

33. B. Zhao, Z. Sun, H. Guo, Communication: State-to-state dynamics of the Cl + $H_2O \rightarrow$ HCl + OH reaction: Energy flow into reaction coordinate and transition-state control of product energy disposal. *J. Chem. Phys.* **142**, 241101 (2015).

34. B. Zhao, H. Guo, Modulations of transition-state control of state-to-state dynamics in the F + $H_2O \rightarrow$HF+OH reaction. *J. Phys. Chem. Lett.* **6**, 676–680 (2015).

35. R. Ellerbrock, U. Manthe, Communication: Reactivity borrowing in the mode selective chemistry of H + $CHD_3 \rightarrow H_2$ + CD_3. *J. Chem. Phys.* **147**, 241104 (2017).

36. R. Ellerbrock, U. Manthe, Full-dimensional quantum dynamics calculations for H + $CHD_3 \rightarrow H_2$ + CD_3: The effect of multiple vibrational excitations. *J. Chem. Phys.* **148**, 224303 (2018).

37. R. Ellerbrock, U. Manthe, Natural reaction channels in H + $CHD_3 \rightarrow H_2$ + CD_3. *Faraday Discuss.* **212**, 217–235 (2018).

38. R. Schinke, *Photodissociation Dynamics* (Cambridge Univ. Press, 1999).

39. W. H. Miller, Quantum mechanical transition state theory and a new semiclassical model for reaction rate constants. *J. Chem. Phys.* **61**, 1823–1834 (1974).

40. W. H. Miller, S. D. Schwartz, J. W. Tromp, Quantum mechanical rate constants for bimolecular reactions. *J. Chem. Phys.* **79**, 4889–4898 (1983).

41. U. Manthe, W. H. Miller, The cumulative reaction probability as eigenvalue problem. *J. Chem. Phys.* **99**, 3411–3419 (1993).

42. R. Welsch, F. Huarte-Larrañaga, U. Manthe, State-to-state reaction probabilities within the quantum transition state framework. *J. Chem. Phys.* **136**, 064117 (2012).

43. H.-D. Meyer, U. Manthe, L. S. Cederbaum, The multi-configurational time-dependent Hartree approach. *Chem. Phys. Lett.* **165**, 73–78 (1990).

44. U. Manthe, H.-D. Meyer, L. S. Cederbaum, Wave-packet dynamics within the multiconfiguration Hartree framework: General aspects and application to NOCl. *J. Chem. Phys.* **97**, 3199–3213 (1992).

45. H. Wang, M. Thoss, Multilayer formulation of the multiconfiguration time-dependent Hartree theory. *J. Chem. Phys.* **119**, 1289–1299 (2003).

46. U. Manthe, A multilayer multiconfigurational time-dependent Hartree approach for quantum dynamics on general potential energy surfaces. *J. Chem. Phys.* **128**, 164116 (2008).

47. U. Manthe, The state averaged multiconfigurational time-dependent Hartree approach: Vibrational state and reaction rate calculations. *J. Chem. Phys.* **128**, 064108 (2008).

48. U. Manthe, A time-dependent discrete variable representation for (multiconfiguration) Hartree methods. *J. Chem. Phys.* **105**, 6989–6994 (1996).

49. U. Manthe, Layered discrete variable representations and their application within the multiconfigurational time-dependent Hartree approach. *J. Chem. Phys.* **130**, 054109 (2009).

50. X. Xu, J. Chen, D. H. Zhang, Global potential energy surface for the H + $CH_4 \rightarrow H_2 + CH_3$ reaction using neural networks. *Chin. J. Chem. Phys.* **27**, 373 (2014).

51. R. Welsch, U. Manthe, Full-dimensional and reduced-dimensional calculations of initial state-selected reaction probabilities studying the H + $CH_4 \rightarrow H_2 + CH_3$ reaction on a neural network PES. *J. Chem. Phys.* **142**, 064309 (2015).

52. G. Schiffel, U. Manthe, Quantum dynamics of the H + $CH_4 \rightarrow H_2 + CH_3$ reaction in curvilinear coordinates: Full-dimensional and reduced dimensional calculations of reaction rates. *J. Chem. Phys.* **132**, 084103 (2010).

53. G. Schiffel, U. Manthe, A transition state view on reactive scattering: Initial state-selected reaction probabilities for the H + $CH_4 \rightarrow H_2 + CH_3$ reaction studied in full dimensionality. *J. Chem. Phys.* **133**, 174124 (2010).

54. R. Welsch, U. Manthe, The role of the transition state in polyatomic reactions: Initial state-selected reaction probabilities of the H + $CH_4 \rightarrow H_2 + CH_3$ reaction. *J. Chem. Phys.* **141**, 174313 (2014).

55. U. Manthe, R. Welsch, Correlation functions for fully or partially state-resolved reactive scattering calculations. *J. Chem. Phys.* **140**, 244113 (2014).

56. C. Evenhuis, G. Nyman, U. Manthe, Quantum dynamics of the $CH-3$ fragment: A curvilinear coordinate system and kinetic energy operators. *J. Chem. Phys.* **127**, 144302 (2007).

57. J. Palma, D. C. Clary, A quantum model Hamiltonian to treat reactions of the type $X+YCZ_3 \rightarrow XY+CZ_3$: Application to $O(^3P)+CH_4 \rightarrow OH+CH_3$. *J. Chem. Phys.* **112**, 1859 (2000).
